# Bad manners in the Emergency Department: Incivility among doctors

**DOI:** 10.1371/journal.pone.0194933

**Published:** 2018-03-29

**Authors:** Karsten Klingberg, Khaled Gadelhak, Sabrina N. Jegerlehner, Adam D. Brown, Aristomenis K. Exadaktylos, David S. Srivastava

**Affiliations:** 1 Emergency Department, Bern University Hospital, Bern, Switzerland; 2 Emergency Department, St Vincent’s University Hospital, Dublin, Ireland; 3 Accident & Emergency, Barts Health NHS Trust, London, United Kingdom; 4 Department of Psychology, Sarah Lawrence College, New York, United States; 5 Department of Psychiatry, New York University School of Medicine, New York, United States; University of Colorado Denver, UNITED STATES

## Abstract

**Introduction:**

Negative workplace behaviour, especially negative communication is a recognised problem in many organisations and is known to have serious impact on workplace performance, productivity and personal wellbeing. Emergency Departments (ED) can be high stress environments in which communication and perceptions of respect between physicians and other staff may underlie individual functioning. We conducted a study to estimate the influence of incivility (ICV) among physicians in the ED.

**Methods:**

We developed an online survey to assess workplace incivility in the ED. We focussed on frequency, origin, reasons and situations where ICV was reported. To measure the levels and the potential influence of ICV on psychological safety, social stress and personal wellbeing we correlated our questionnaire to standard psychological scales. Statistical analysis included Students t-test, chi squared distribution and Pearson correlation coefficient.

**Results:**

We invited all seventy-seven ED physicians to participate in our survey. Among those that completed (n = 50, 65%) the survey, 9% of ED physicians reported frequent (1/week) and 38% occasional (1/month) incidents of ICV. 28% of physicians reported experiencing ICV once per quarter and 21% reported a frequency of only once per year, no physician reported ICV on a daily basis. Levels of ICV were significantly higher in interactions with specialists from outside then within the ED (*p*<0.01).

ICV was perceived particularly during critical situations. Our findings showed a significant correlation between internal (within the ED team) ICV and psychological safety. To ED physicians internal ICV was associated with lower levels of psychological safety (*p*<0.01). ICV displayed from sources outside the ED team was not associated with psychological safety, but we found a significant influence of external ICV on personal irritability and reduced wellbeing (*p*<0.01).

**Discussion:**

The incidence of incivility was high among the ED physicians. Although this was a small sample, the association between workplace ICV and psychological safety, personal irritation as well personal comfort suggests that ICV may be an important variable underlying ED team performance. These findings further underscore the need to foster a culture of respect and good communication between departments, as levels of ICV were highest with physicians from outside the ED. Future research would benefit from examining strategies to prevent and reduce ICV and identify reasons for personal variation in perception of ICV. During critical situations and in general collaboration with specialists, awareness of ICV and countermeasures are important to avoid decreased performance and negative impact on staff and patient.

## Introduction

Emergency Departments (ED’s) are complex work environments. Staff is often required to respond to complex urgent medical issues with varying degrees of background information on the patient arriving in the ED. Furthermore, it has been reported that working in an ED can be demanding as staff report high levels of stress [1], long work-hours [2], high work volume, and lack of role clarity between ED staff and other departments [3]. As such, studies show that ED physicians report elevated rates of depression, anxiety, burnout, absenteeism, high turnover, and early retirement [4].

Communication issues and task interruptions are among the most important factors associated with stress in the ED [5]. ICV, as one aspect of a wider range of negative communication, has been defined as a violation of norms in social interactions, shown as disregard of co-workers, causing situations of disrespect, conflict, and stress [6]. Though ICV may be seen as an ordinary part of one’s work environment, ICV has been associated with a wide range of serious negative outcomes throughout the workplace [7].

Previous work has shown that ICV is common in the workplace. For example, a large study of 5288 individuals in 70 different organisations including hospitals revealed that 10% of the employees reported being exposed to negative behaviour during the last 6 months [8]. Additionally, research conducted in 17 industries showed that 80% of the participants who have been victim of incivility lost work time worrying about the incident and felt that their commitment to the organization declined. Work performance including work effort and quality was intentionally dropped by 40–50%, and 25% of the employees admitted that they took their frustration out on to customers [9]. On an individual level, experimental studies revealed that exposure to ICV subsequently led to impairment in cognitive performance in domains such as, memory and attention as well as decreased cooperation and willingness to help others [10].

A study from the National Health Service (NHS) in the United Kingdom (UK) found that up to 31% of physicians are exposed to ICV multiple times per week, and up to 40% reported that ICV had a moderately to severe daily impact on their work [11]. Research in non-profit hospitals in the US has shown negative effects of ICV on the quality of life with feelings of frustration and stress in 90% of the affected. Additionally, the critical effect on concentration, collaboration and communication was interpreted to be linked to 71% of medical errors and even to increased mortality in 27% [12]. A survey on ICV among nurses calculated the financial impact of incivility in a loss of productivity of more than 11.000 USD per nurse per year [13]. Altogether, this underlines that not only personal wellbeing and job satisfaction, but also work performance measured in motivation and general productivity is affected by ICV [14].

ED’s may be particularly vulnerable for ICV, as ED’s are often a central connection point for the hospital with multiple interactions between ED physicians and a variety of medical specialities and different types of staff. Communication within the ED and between departments is required for patient care and and has been shown to be crucial in the wellbeing of patients and medical staff [5].

The aim of our study was to assess the influence of ICV on the team of ED physicians.

## Materials and methods

The survey was conducted in the ED of the University Hospital in Bern, Switzerland. The ED team treats more than 46’000 patients per year. During 24/7 all clinical specialities are available.

In three focus group meetings, 10 junior and 10 senior ED physicians were interviewed by an organizational psychologist, to evaluate key points of ICV. Based on these findings, we constructed our online survey. Our research question focused on frequency, origin, reasons and situations of ICV. In order to measure the level and the association of ICV on personal safety, social stress and personal wellbeing the questionnaire was adjusted to standard psychological scales.

The intensity of ICV was measured with a series of items based on the definitions and concepts of Cortina et al. which have been widely used and validated in occupational settings [15].

The irritation scale after Mohr et al. was used to assess emotional and cognitive strain in the ED. The scale has been used in many contexts and in previous studies in occupational health assessments. It has been shown to be a predictor of workplace satisfaction and commitment [16].

The social stressor scale after Zapf et al. estimated work related stress. The scale includes items regarding possible occupational and personal consequences associated with stress [17].

Psychological safety, measured with the scale after Edmondson et al., is positively associated with the team’s ability to learn and innovate [18].

According to the results of our preliminary interview session we clustered the results on the psychological scales based on the origin of ICV from inside the ED team versus outside the ED team. All physicians (n = 77) working in the ED of the University Hospital Bern, Switzerland, were invited to our survey. We sent a link to an online survey via Email and several reminders were sent out to ensure compliance.

Statistical analysis including Students t-test, chi squared distribution and Pearson correlation coefficient was performed using SPSS Version 19. The data is given as absolute numbers [mean value (M), standard deviation (SD)] or as percentage. The study was performed according to Swiss law. No patient related data was used. The participating physicians gave their consent to analyse and publish their anonymised data.

## Results

**Fig 1:**
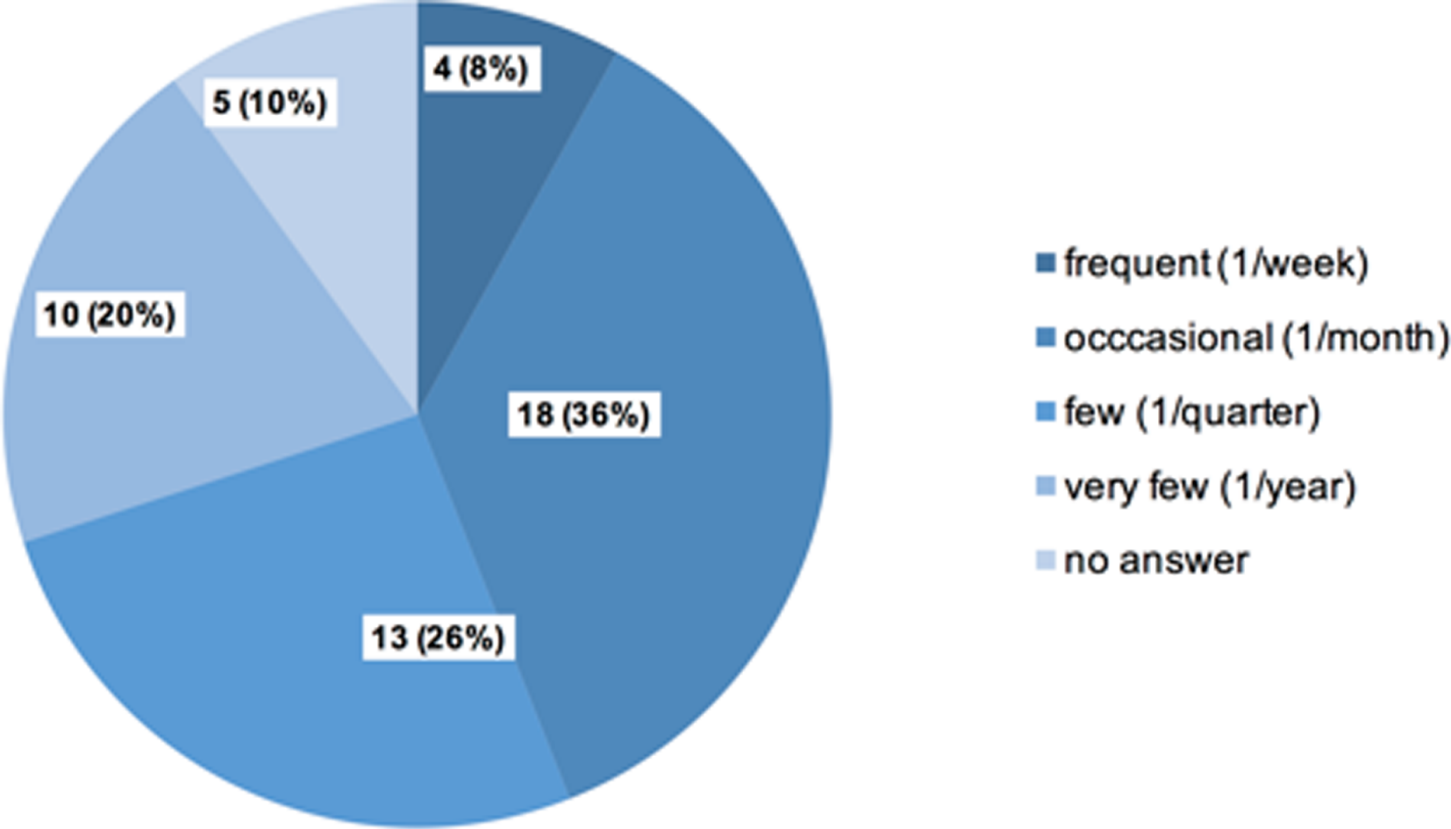
Frequency of incivility perceived by ED physicians. n (%).

50 out of 77 ED physicians completed part of the survey (response rate 65%). There were 25 female and 19 male participants of all grades (6 did not answer). Frequency of ICV was reported as follows: 4 physicians (8%) reported frequent ICV (1/week) and 18 (36%) occasional (1/month) incidents of ICV, 13 (26%) reported exposure to ICV once per quarter and 10 (20%) reported a frequency of only once per year. No physician reported ICV on a daily base, 5 (10%) did not answer (Fig 1).

**Fig 2:**
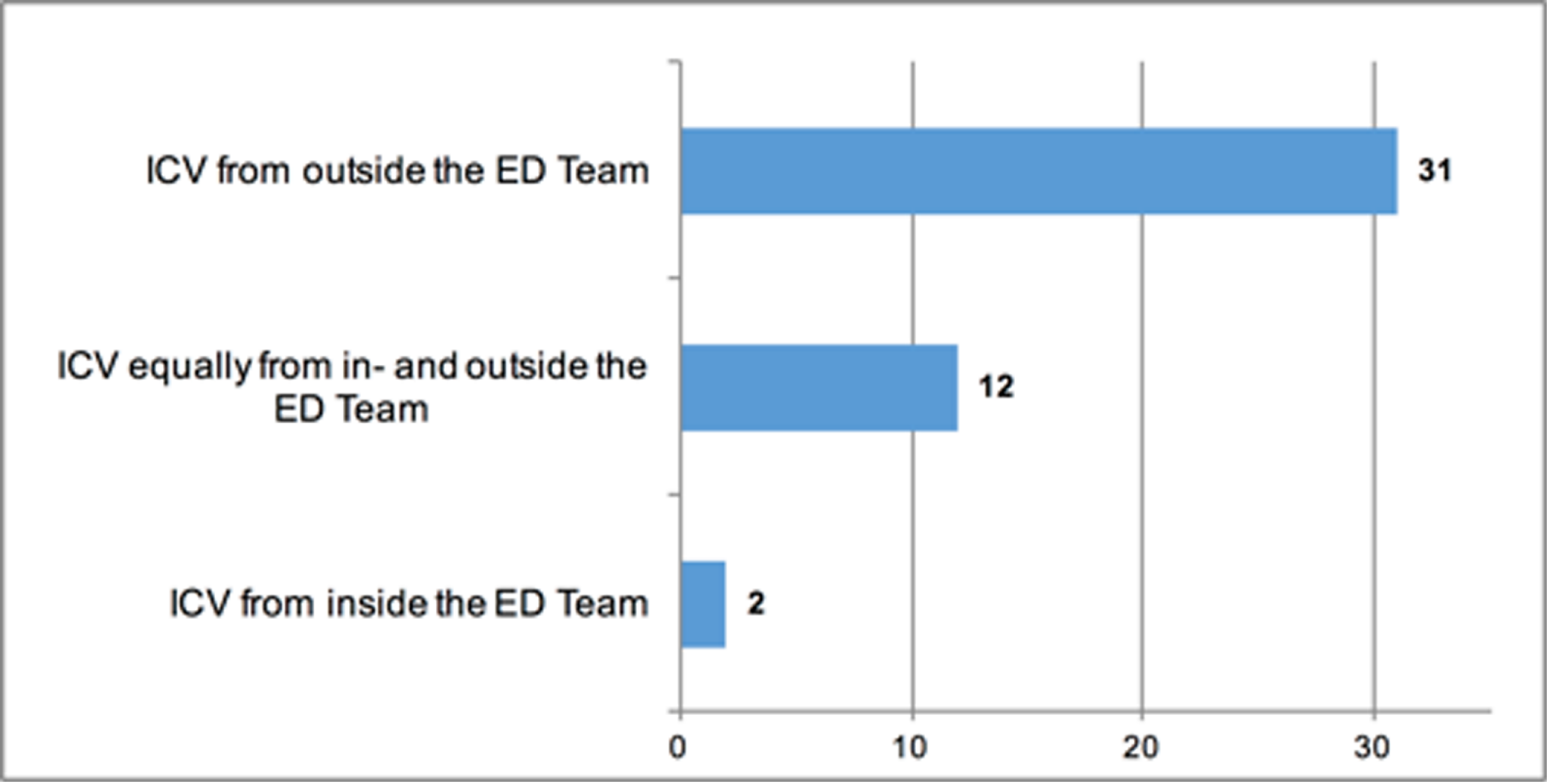
Origin of perceived incivility as by ED physicians. (n = 45, no answer = 5).

ED physicians reported that they saw the main origin of ICV in relation to other specialities, 31 (62%). 12 (24%) ED physicians reported equally distributed ICV in- and outside the ED but only one ED physician (2%) perceived greater ICV within the ED than outside of the ED, 6 ED physicians (12%) did not answer this question (Fig 2).

**Fig 3:**
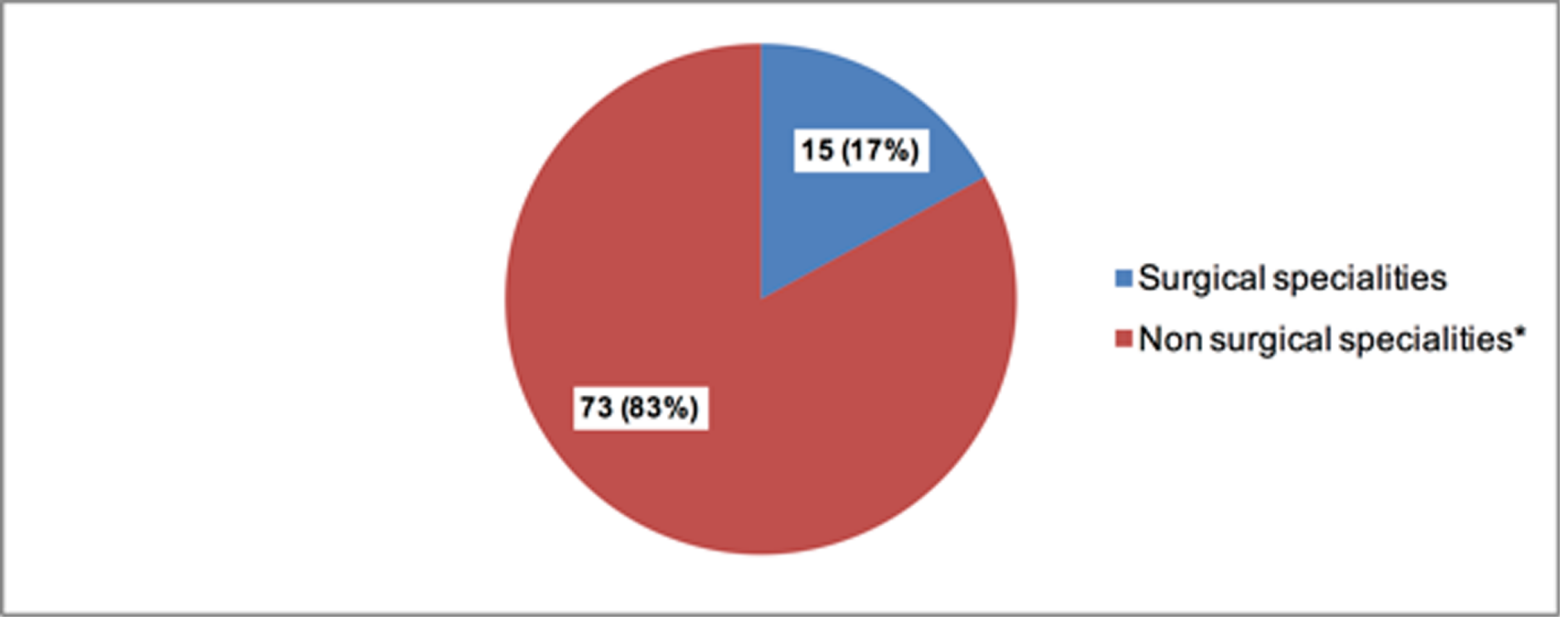
Perpetrators of ICV from outside the ED team clustered into surgical and non-surgical specialities*. (n (%), up to 3 responses possible). *Intensive Care, Gastroenterology, Cardiology, Anaesthesiology, Psychiatry, Radiology.

Asking to choose the originating departments of external ICV (maximum three choices), we clustered results into non-surgical and surgical departments in order to to avoid blame. Non-surgical specialities (Intensive Care, Gastroenterology, Cardiology, Anaesthesiology, Psychiatry, Radiology) were named in 73 (83%) versus only 15 (17%) in surgical fields of all nominations (Fig 3).

**Fig 4:**
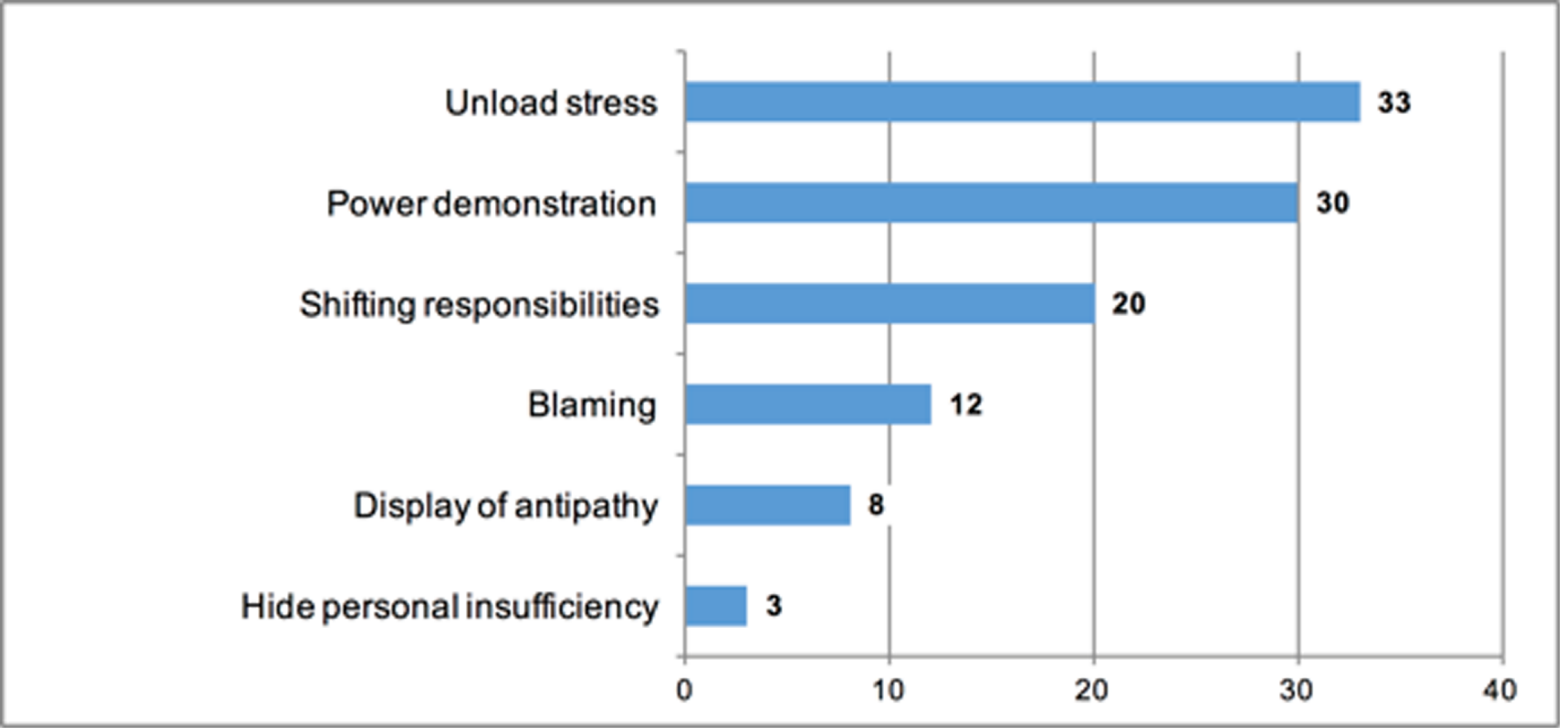
Subjective interpretation of perceived ICV. (n = 106, multiple answers possible).

The perceived reasons for ICV (multiple choices were allowed) were described as power demonstration (30; 28%) or to unload stress (33; 31%) in 63 (59%) of the answers. Whereas, blame (n = 12; 11%) or shifting responsibility (n = 20; 19%) was selected in 22 (21%) of the answers (Fig 4).

**Fig 5:**
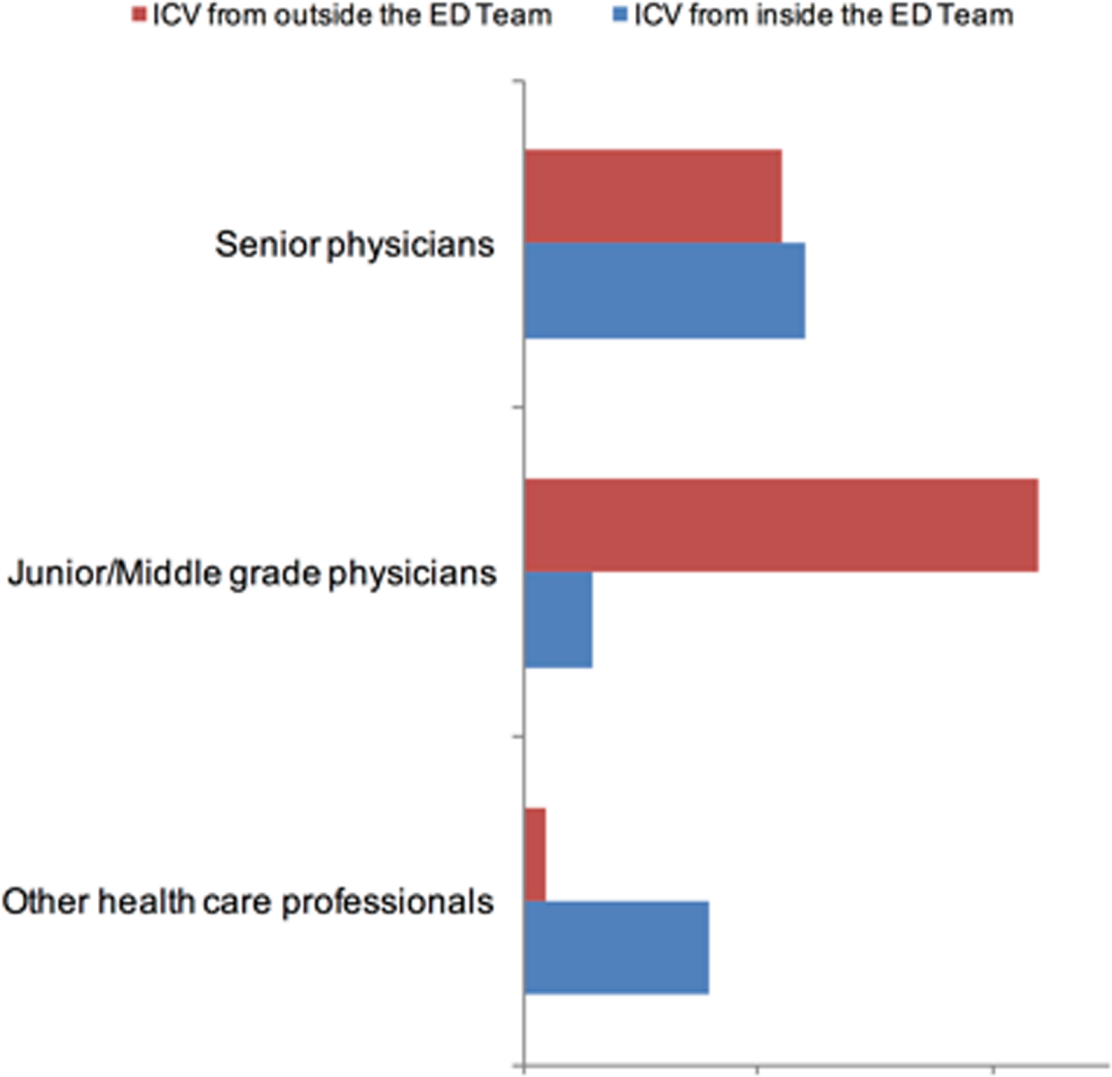
Hierarchic origin of ICV perceived by the ED physicians comparing inside and outside the ED team.

ICV from outside the ED Team originated mostly between peers. Within the ED individuals reported ICV in relation to hierarchic order and between doctors and nurses (Fig 5).

**Fig 6:**
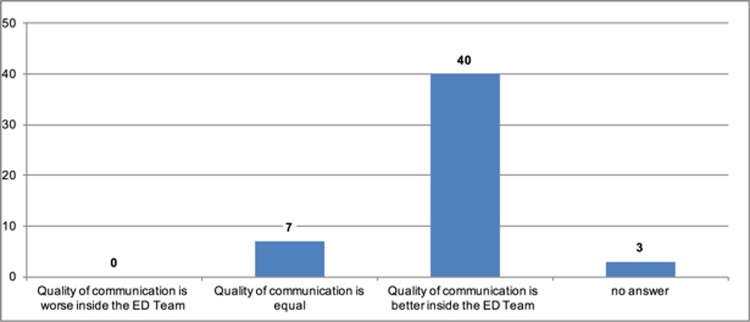
Quality of communication compared inside and outside the ED team.

Participants reported that communication was better (n = 40; 80%) or equal (n = 7; 14%) within the ED compared to communication with physicians outside of the ED. No one reported communication was better with physicians outside of the ED (Fig 6).

**Fig 7:**
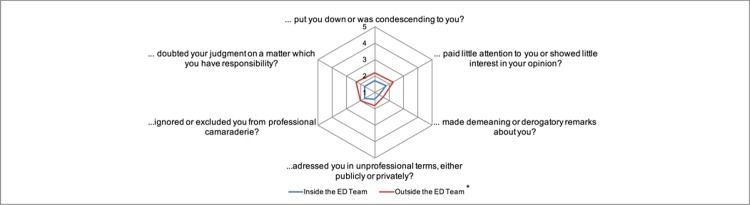
Perceived intensity of ICV after Cortina et al.[15] comparing inside and outside the ED team. We asked the physicians how often they perceived the following within the last year, range: 1 = low to 6 = high (n = 47 no answer = 3), *(*p*<0.01).

ED physicians reported significantly higher Levels of ICV [15] in contact with other specialties (M = 2.02, SD = 0.71) than with the ED team (M = 1.61, SD = 0.48) (t-Test: *p*<0.01) (Fig 7).

**Fig 8:**
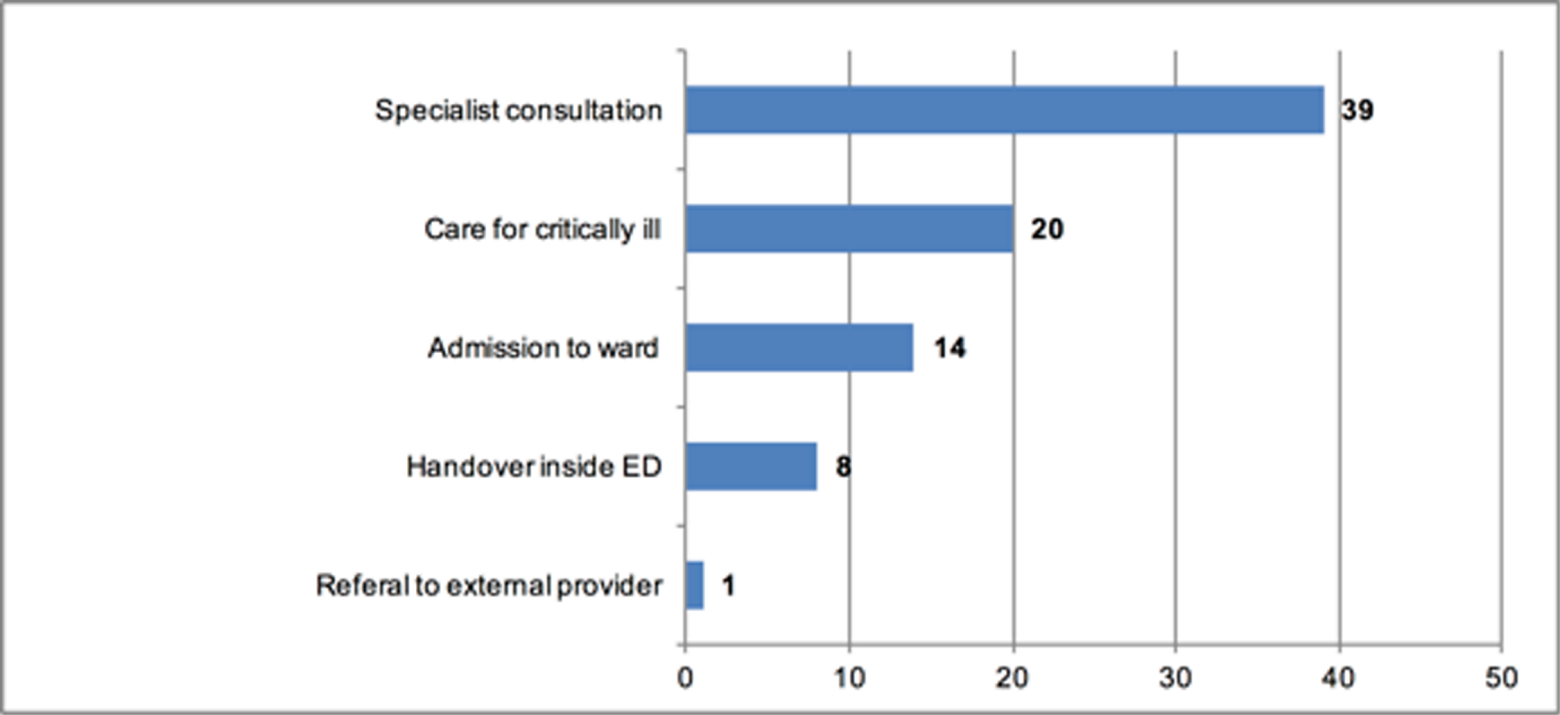
Situations in which ICV occurred in the ED. (n = 82, multiple answers possible).

The highest incidence of ICV was reported during specialist consultations or referrals, and even more importantly during care of critically ill patients (Fig 8).

Results of our correlation analysis showed no significant interrelation between the frequency of ICV and the degree of experienced social stressors (r = 0.16), reduced personal wellbeing (r = -0.43) or psychological safety (r = -0.19).

Higher social stressors from inside the ED team showed a significant correlation with individual psychological safety (r = -0.45, *p*<0.01). Psychological safety was also affected by higher levels of internal ICV (r = -0.50, *p*<0.01).

On the other hand, levels of ICV displayed from external sources was not significantly correlated with psychological safety (r = -0.20), but we found a significant correlation between personal irritability and reduced wellbeing (r = -0,38, *p*<0.01).

## Discussion

The response rate of 65% in our survey is similar to previous studies and designs in this context and underlines the relevance of this topic [19]. Almost every second physician (47%) claimed frequent or occasional ICV in the ED. This prevalence seems to be equal or less frequent in comparison to current literature and remains unsatisfactory high [11, 20].

Participants reported stress relation, power demonstration and denying liability as the most relevant reasons for ICV (Fig 4). These items reflect interpersonal and individual levels of interpretation by the receptionist. Awareness of the nature and dynamics of ICV is crucial in a high-pressure medical environment, such as an ED. This seems to be even more relevant as the results revealed that in critical conditions, e.g. during collaboration with many specialists in the resuscitation room a high rate of ICV was experienced by the ED physicians. In this particular situation, close and effective collaboration between all specialities is paramount to provide best medical practice for patients. The use of Crew Resource Management tools and implementation of inter-professional trainings has a proven effect to minimise errors and should also address communication skills to reduce ICV [20–22].

Regarding the quality of communication within the ED staff, it appears that among ED physicians there seems to be an energetic communication level between the team members, a feature of well performing teams in different industries [23]. Another more obvious interpretation of the good communication within the team is the shared situation and accountability of the ED physicians.

As already in other studies reported [24], we found some ICV originating from other healthcare professionals inside the ED. This might be explained by different goals and objectives regarding patient care and can be addressed by inter-professional trainings and team building measures [25].

Our survey revealed the greatest difference concerning ICV in comparison between communications within the ED versus communication outside the ED. Highest rates of ICV were reported in contact with physicians from other departments. Even in this study we use terms like “inside” and “outside” in order to clarify our results, but we are aware of the risk of generalising the findings to an organisational level. The fact that cooperation with surgical fields had a much lower rate in perceived ICV by ED physicians is in strong contrast to previous studies where surgical specialities were stated to be the main initiator of ICV [26, 27]. Similar findings with high amounts of ICV originating from non-surgical specialities were described by Bradley et al. but neither our data nor Bradley et al. provide an explanation of this fact[11].

ICV is generally known to affect personal wellbeing, a factor that influences team work and performance [14]. Our findings showed two major results, first a significant correlation between level of internal ICV and psychological safety, which is a key factor for innovation and learning in teams [9] and second a significant correlation between levels of external ICV and irritation/ personal wellbeing, which can have a negative impact on workplace satisfaction and commitment. Why ICV originating from within the team affects the recipient on a different level than from outside the team cannot be explained in our study and warrants further research. There is a growing number of countermeasures against workplace incivility which may be modified and tested in this setting [12, 28]. Awareness and understanding of external and internal factors that lead to ICV should focus on support and development on a personal base [29].

Interventions on a departmental level targeting processes like “zero tolerance policies” or specific training for staff including leadership programs can improve workplace behaviour and reduce negative effects like depression and burnout [30].

ED management needs to take action in order to reduce ICV as the performance of ED teams has direct influence on patient outcomes [24]. Future work would benefit from examining the outcomes of interventions against ICV on quality of care, patient safety and staff satisfaction [31].

Time to behave, Doctor!

### Limitations

Our survey was performed as a single centre study only among physicians in the ED despite the fact that ED teams are strongly built on interdisciplinary staff. Small sample size limits the interpretation of our results. Inclusion of other ED professionals and departments to integrate the overall importance of ICV was not our primary goal but it should be addressed by further studies. We report results and views only from subjective perspectives with the constraint that several perceived events can only be displayed as a scaled down and maybe more general answer on the survey.

Furthermore, we did not investigate origins of ICV caused by the recipient himself as this was not a concern during the preliminary interviews and report of this situation is expected to be unreliable. This constricts this interpretation of the causes of ICV to a certain degree. A probable selection bias in this self-reported study design cannot be excluded. Those affected by ICV may not want to participate, or more likely, it was predominantly those who were affected who participated in the survey. We tried to minimise this with the use of standardised psychological scales, which proved that at the time of the survey team members were neither stressed nor emotionally strained.

## Conclusions

1. Awareness is a relevant step to challenge ICV in the ED. It should not be an accepted part of workplace culture.
2. ICV occurs mostly in interaction with members of other teams and during critical situations.
3. ICV from outside the ED team is correlated with higher irritability and reduced wellbeing of the affected individual.
4. Social stressors and higher levels of ICV inside the ED team affect psychological safety. A key factor for innovation and forward thinking in teams.

## Supporting information

S1 File Questionnaire. (PDF)

S2 File Dataset. (PDF)

S3 File Questionnaire translation. (PDF)

## References

[1] Lepnurm R, Lockhart WS, Keegan D. A measure of daily distress in practising medicine. The Canadian Journal of Psychiatry. 2009;54(3):170–80. doi: 10.1177/070674370905400305 19321021 10.1177/070674370905400305

[2] Whitehead DC, Thomas H, Slapper DR. A rational approach to shift work in emergency medicine. Annals of emergency medicine. 1992;21(10):1250–8. 1416310 10.1016/s0196-0644(05)81758-5

[3] Ummenhofer W, Amsler F, Sutter PM, Martina B, Martin J, Scheidegger D. Team performance in the emergency room: assessment of inter-disciplinary attitudes. Resuscitation. 2001;49(1):39–46. 11334690 10.1016/s0300-9572(00)00304-x

[4] Bragard I, Dupuis G, Fleet R. Quality of work life, burnout, and stress in emergency department physicians: a qualitative review. European Journal of Emergency Medicine. 2015;22(4):227–34. doi: 10.1097/MEJ.0000000000000194 25093897 10.1097/MEJ.0000000000000194

[5] Spencer R, Coiera E, Logan P. Variation in communication loads on clinical staff in the emergency department. Annals of emergency medicine. 2004;44(3):268–73. doi: 10.1016/S0196064404004147 15332070 10.1016/j.annemergmed.2004.04.006

[6] Andersson LM, Pearson CM. Tit for tat? The spiraling effect of incivility in the workplace. Academy of management review. 1999;24(3):452–71.

[7] Pearson CM, Porath CL. On the nature, consequences and remedies of workplace incivility: No time for “nice”? Think again. The Academy of Management Executive. 2005;19(1):7–18.

[8] Hoel H, Cooper CL. Destructive conflict and bullying at work: Manchester School of Management, UMIST Manchester; 2000.

[9] Porath C, Pearson C. The price of incivility. Harvard business review. 2013;91(1–2):114–21. 23390745

[10] Porath CL, Erez A. Does rudeness really matter? The effects of rudeness on task performance and helpfulness. Academy of Management Journal. 2007;50(5):1181–97.

[11] Bradley V, Liddle S, Shaw R, Savage E, Rabbitts R, Trim C, et al Sticks and stones: investigating rude, dismissive and aggressive communication between doctors. Clinical Medicine. 2015;15(6):541–5. doi: 10.7861/clinmedicine.15-6-541 26621942 10.7861/clinmedicine.15-6-541 PMC4953255

[12] Rosenstein AH, O'Daniel M. A survey of the impact of disruptive behaviors and communication defects on patient safety. The Joint Commission Journal on Quality and Patient Safety. 2008;34(8):464–71. 18714748 10.1016/s1553-7250(08)34058-6

[13] Lewis PS, Malecha A. The impact of workplace incivility on the work environment, manager skill, and productivity. Journal of Nursing Administration. 2011;41(1):41–7. doi: 10.1097/NNA.0b013e3182002a4c 21157243 10.1097/NNA.0b013e3182002a4c

[14] Burnes B, Pope R. Negative behaviours in the workplace: A study of two Primary Care Trusts in the NHS. International Journal of Public Sector Management. 2007;20(4):285–303.

[15] Cortina LM, Magley VJ, Williams JH, Langhout RD. Incivility in the workplace: incidence and impact. Journal of occupational health psychology. 2001;6(1):64 11199258

[16] Mohr G, Rigotti T, Müller A. Instrumente der Arbeits-und Organisationspsychologie. Irritation œ ein Instrument zur Erfassung psychischer Beanspruchung im Arbeitskontext. Skalen-und Itemparameter aus 15 Studien. Zeitschrift für Arbeits-und Organisationspsychologie. 2005;49(1):44–8.

[17] Zapf D, Frese M. Soziale Stressoren am Arbeitsplatz. Psychischer Streβ am Arbeitsplatz. 1991:168–84.

[18] Edmondson A. Psychological safety and learning behavior in work teams. Administrative science quarterly. 1999;44(2):350–83.

[19] Quine L. Workplace bullying, psychological distress, and job satisfaction in junior doctors. Cambridge Quarterly of Healthcare Ethics. 2003;12(1):91–101. 12625206 10.1017/s0963180103121111

[20] Hughes KM, Benenson RS, Krichten AE, Clancy KD, Ryan JP, Hammond C. A crew resource management program tailored to trauma resuscitation improves team behavior and communication. Journal of the American College of Surgeons. 2014;219(3):545–51. doi: 10.1016/j.jamcollsurg.2014.03.049 25026871 10.1016/j.jamcollsurg.2014.03.049

[21] Riskin A, Erez A, Foulk TA, Kugelman A, Gover A, Shoris I, et al The impact of rudeness on medical team performance: a randomized trial. Pediatrics. 2015;136(3):487–95. doi: 10.1542/peds.2015-1385 26260718 10.1542/peds.2015-1385

[22] Flin R, Maran N. Identifying and training non-technical skills for teams in acute medicine. Quality and Safety in Health care. 2004;13(suppl 1):i80–i4. 15465960 10.1136/qshc.2004.009993 PMC1765790

[23] Pentland A. The new science of building great teams. Harvard Business Review. 2012;90(4):60–9.23074865

[24] Rosenstein AH, Naylor B. Incidence and impact of physician and nurse disruptive behaviors in the emergency department. The Journal of emergency medicine. 2012;43(1):139–48. doi: 10.1016/j.jemermed.2011.01.019 21421291 10.1016/j.jemermed.2011.01.019

[25] Baggs JG, Schmitt MH, Mushlin AI, Mitchell PH, Eldredge DH, Oakes D, et al Association between nurse-physician collaboration and patient outcomes in three intensive care units. Critical care medicine. 1999;27(9):1991–8. 10507630 10.1097/00003246-199909000-00045

[26] Roberts NK, Dorsey JK, Wold B. Unprofessional behavior by specialty: A qualitative analysis of six years of student perceptions of medical school faculty. Medical teacher. 2014;36(7):621–5. doi: 10.3109/0142159X.2014.899690 24787525 10.3109/0142159X.2014.899690

[27] Goettler CE, Butler TS, Shackleford P, Rotondo MF. Physician Behavior: Not Ready for ‘Never'land. The American Surgeon. 2011;77(12):1600–5. 22273216

[28] Porath C. An antidote to incivility. Harvard business review. 2016;94(4):22.

[29] Rosenstein A. Addressing the causes and consequences of disruptive behaviors in the healthcare setting. Journal of Psychology and Clinic Psychiatry. 2015;3(3):00136.

[30] Rosenstein AH. BAD MEDICINE. Risk Management. 2013;60(10):38.

[31] Rosenstein AH. Physician disruptive behaviors: Five year progress report. World journal of clinical cases. 2015;3(11):930 doi: 10.12998/wjcc.v3.i11.930 26601095 10.12998/wjcc.v3.i11.930 PMC4644894

