# Supplementary material for: Bad manners in the Emergency Department: Incivility among doctors
Source: PLoS One. 2018 Mar 29;13(3):e0194933. doi: 10.1371/journal.pone.0194933 (PMC5875803; doi:10.1371/journal.pone.0194933)
Supplement: S1 File — (PDF) [file pone.0194933.s001.pdf]

Feindselige Kommunikation unter Ärztinnen und Ärzten

Gegenseitiger Umgang auf dem UNZ

Wie beurteilen Sie die folgenden Aspekte?

	trifft gar nicht zu	trifft eher nicht zu	teils - teils	trifft eher zu	trifft sehr zu	nicht beurteilbar
1. Alle trauen sich, Probleme und heikle Themen anzusprechen.	○	○	○	○	○	○
2. Niemand würde absichtlich meine Bemühungen untergraben.	○	○	○	○	○	○
3. Wenn jemand einen Fehler macht, wird dies oft gegen ihn verwendet.	○	○	○	○	○	○
4. Manche Personen weisen andere wegen ihrer Andersartigkeit zurück.	○	○	○	○	○	○
5. Andere schätzen und nutzen meine speziellen Fähigkeiten und Talente.	○	○	○	○	○	○
6. Es ist schwierig, andere um Hilfe zu bitten.	○	○	○	○	○	○
7. Man kann auf der zwischenmenschlichen Ebene auch mal ein Risiko eingehen.	○	○	○	○	○	○
8. Ich werde von meinen Vorgesetzten dazu ermutigt, eigeninitiativ zu handeln.	○	○	○	○	○	○
9. Ich werde von meinen Vorgesetzten jeweils nach meinen Ideen und Anregungen gefragt.	○	○	○	○	○	○
10. Meine Meinung ist aus Sicht meiner Vorgesetzten oft weniger wert als ihre eigene Meinung.	○	○	○	○	○	○
11. Meine Vorgesetzten bringen zum Ausdruck, dass sie meine Ideen und Meinungen schätzen.	○	○	○	○	○	○

Bemerkungen

Feindselige Kommunikation unter Ärztinnen und Ärzten

Wie sehr treffen die Aussagen auf die Zusammenarbeit mit ärztlichen Kolleg/innen zu?

Innerhalb des UNZ?

	trifft nicht zu	trifft wenig zu	trifft ziemlich zu	tritt zu	nicht beurteilbar
1. Mit einigen Kollegen hat man oft Streit.	○	○	○	○	○
2. Einige Kollegen sind unangenehme Mitarbeiter.	○	○	○	○	○
3. Hier wird man wegen jeder Kleinigkeit gleich fertiggemacht.	○	○	○	○	○
4. Hier gibt es immer nur Kritik, was man gut macht sieht keiner.	○	○	○	○	○
5. Man muss mit unfreundlichen Leuten zusammenarbeiten.	○	○	○	○	○
6. Man muss mit Leuten zusammenarbeiten, die keinen Spaß verstehen.	○	○	○	○	○
7. Es gibt Schwierigkeiten bei der Abstimmung mit Kollegen.	○	○	○	○	○
8. Man muss ausbaden, was die anderen falsch machen.	○	○	○	○	○
9. Wenn ein Fehler passiert, finden manche KollegInnen ihn immer bei mir, nie bei sich.	○	○	○	○	○

Bemerkungen

Mit anderen Kliniken und Abteilungen?

	trifft nicht zu	trifft wenig zu	trifft ziemlich zu	tritt zu	nicht beurteilbar
1. Mit einigen Kollegen hat man oft Streit.	○	○	○	○	○
2. Einige Kollegen sind unangenehme Mitarbeiter.	○	○	○	○	○
3. Hier wird man wegen jeder Kleinigkeit gleich fertiggemacht.	○	○	○	○	○
4. Hier gibt es immer nur Kritik, was man gut macht sieht keiner.	○	○	○	○	○
5. Man muss mit unfreundlichen Leuten zusammenarbeiten.	○	○	○	○	○
6. Man muss mit Leuten zusammenarbeiten, die keinen Spaß verstehen.	○	○	○	○	○
7. Es gibt Schwierigkeiten bei der Abstimmung mit Kollegen.	○	○	○	○	○
8. Man muss ausbaden, was die anderen falsch machen.	○	○	○	○	○
9. Wenn ein Fehler passiert, finden manche KollegInnen ihn immer bei mir, nie bei sich.	○	○	○	○	○

Bemerkungen

Feindselige Kommunikation unter Ärztinnen und Ärzten

Im vergangenen letzten halben Jahr auf dem UNZ, wie oft kam es vor, dass ein/e ärztlicheKolleg/in...

...des UNZ...

	niemals	selten	manchmal	oft	meistens	nicht beurteilbar
a) Sie blöd dastehen liess oder herablassend zu Ihnen war?	○	○	○	○	○	○
b) Ihrer Aussage kaum Beachtung schenkte oder kaum Interesse an Ihrer Meinung zeigte?	○	○	○	○	○	○
c) erniedrigende oder abschätzige Bemerkungen über Sie machte?	○	○	○	○	○	○
d) Sie in unprofessioneller Weise anredete?	○	○	○	○	○	○
e) Sie überging oder von Gesprächen mit anderen Kollegen ausschloss?	○	○	○	○	○	○
f) Ihre Beurteilung in Ihrem Zuständigkeitsbereich anzweifelte?	○	○	○	○	○	○

Bemerkungen

... einer anderen Klinik oder Abteilung...

	niemals	selten	manchmal	oft	meistens	nicht beurteilbar
a) Sie blöd dastehen liess oder herablassend zu Ihnen war?	○	○	○	○	○	○
b) Ihrer Aussage kaum Beachtung schenkte oder kaum Interesse an Ihrer Meinung zeigte?	○	○	○	○	○	○
c) erniedrigende oder abschätzige Bemerkungen über Sie machte?	○	○	○	○	○	○
d) Sie in unprofessioneller Weise anredete?	○	○	○	○	○	○
e) Sie überging oder von Gesprächen mit anderen Kollegen ausschloss?	○	○	○	○	○	○
f) Ihre Beurteilung in Ihrem Zuständigkeitsbereich anzweifelte?	○	○	○	○	○	○

Bemerkungen

Feindselige Kommunikation unter Ärztinnen und Ärzten

Eigenes Wohlbefinden

Wie sehr treffen die folgenden Aussagen auf Sie zu?

	trifft völlig zu	trifft grösstenteils zu	trifft etwas zu	trifft mittelmässig zu	trifft wenig zu	trifft grösstenteils nicht zu	trifft überhaupt nicht zu	nicht beurteilbar
Es fällt mir schwer, nach der Arbeit abzuschalten.								
Ich muß auch zu Hause an Schwierigkeiten bei der Arbeit denken.								
Wenn andere mich ansprechen, kommt es vor, dass ich mürrisch reagiere.								
Selbst im Urlaub muss ich manchmal an Probleme bei der Arbeit denken.								
Ich fühle mich ab und zu wie jemand, den man als Nervenbündel bezeichnet.								
Ich bin schnell verärgert.								
Ich reagiere gereizt, obwohl ich es gar nicht will.								
Wenn ich müde von der Arbeit nach Hause komme, bin ich ziemlich nervös.								

Bemerkungen

Feindselige Kommunikation unter Ärztinnen und Ärzten

Häufigkeit, Art, vermuteter Zweck und typische Situationen feindseliger Kommunikation

Wie oft kommt es nach Ihrer Erfahrung im Schnitt auf dem UNZ zu feindseligen Kommunikationen unter ärztlichen Kolleg/innen?

- ○ sehr selten / nie
- ○ selten (etwa 1x im Quartal)
- ○ gelegentlich (etwa 1x pro Monat)
- ○ oft (etwa 1x pro Woche)
- ○ sehr oft (täglich)
- ○ nicht beurteilbar

Im Vergleich zu Ihren persönlichen Erfahren in anderen Kliniken: Wie beurteilen Sie die Qualität der Kommunikation unter Ärzt/innen auf dem UNZ?

- ○ viel schlechter
- ○ eher schlechter
- ○ genau gleich
- ○ eher besser
- ○ viel besser
- ○ nicht beurteilbar

Welchen Zweck erfüllt die feindselige Kommunikation aus Ihrer Sicht hauptsächlich? (auch Mehrfachnennungen möglich)

- ☐ ungezielt eigenen Stress "abladen"
- ☐ UNZ als Sündenbock dastehen lassen wollen
- ☐ Verantwortung abgeben
- ☐ Machtdemonstration
- ☐ Persönliche Abneigung kundtun
- ☐ Nicht beurteilbar
- ☐ Anderes, nämlich: ______________________

In welchen Situationen kommt es hauptsächlich zu feindseligen Kommunikationen? (auch Mehrfachnennungen möglich)

- [ ] in Akutsituationen (z.B. Schockraum)
- [ ] bei Schichtübergaben UNZ-intern
- [ ] Verlegungen auf Abteilungen inselspitalintern
- [ ] Verlegungen nach extern
- [ ] Konsilanfragen / Beizug von Spezialisten
- [ ] Keine Angabe
- [ ] Weitere mögliche Situationen, nämlich:

Von wo geht die feindselige Kommunikation hauptsächlich aus?

- ○ hauptsächlich UNZ-intern
- ○ hauptsächlich von Fremdkliniken
- ○ gleichverteilt, hälftig UNZ-intern, hälftig -extern
- ○ Anders, nämlich:

Feindselige Kommunikation unter Ärztinnen und Ärzten

Hauptsächliche Quellen feindseliger Kommunikation

Von welchen Funktionsträgern geht die feindselige Kommunikation hauptsächlich aus?

	CA / LA	OA	Stv OA	AA	UA	Pflege	keine Angabe
innerhalb UNZ	○	○	○	○	○	○	○
mit anderen Kliniken & Abteilungen	○	○	○	○	○	○	○

Allgemeine Bemerkung oder andere als die aufgeführten Funktionsträger/innen:

Von welchen Fremdkliniken geht am meisten feindselige Kommunikation aus? Geben Sie bitte über die Auswahlliste die drei häufigsten an.

1. am häufigsten von...	
2. am zweithäufigsten von...	
3. am dritthäufigsten von...	

Allgemeine Bemerkung oder andere als die aufgezählten Kliniken, nämlich:

Feindselige Kommunikation unter Ärztinnen und Ärzten

Persönliche Angaben und allgemeine Schlussbemerkung

Die folgenden Angaben dienen einer differenzierten Auswertung. Es wird kein Rückschluss auf einzelne Personen gezogen.

Ihre Funktion

- ◯ CA / LA
- ◯ OA
- ◯ AA / Stv OA
- ◯ UA
- ◯ Keine Angabe

Anzahl Dienstjahre in der aktuellen Funktion im Inselspital

____

Alter

- ◯ < 30 Jahre
- ◯ 30 bis 39 Jahre
- ◯ 40 bis 49 Jahre
- ◯ > 49 Jahre
- ◯ Keine Angabe

Geschlecht

- ◯ Weiblich
- ◯ Männlich
- ◯ Keine Angabe

Allgemeine Schlussbemerkungen zu der Umfrage

____
