# Supplementary material for: Bad manners in the Emergency Department: Incivility among doctors
Source: PLoS One. 2018 Mar 29;13(3):e0194933. doi: 10.1371/journal.pone.0194933 (PMC5875803; doi:10.1371/journal.pone.0194933)
Supplement: S2 File — (PDF) [file pone.0194933.s002.pdf]

Code	PS_1	PS_2	PS_3	PS_4	PS_5	PS_6	PS_7	PS_8	PS_9
1	5	5	1	1	5	1	4	5	5
2	5	5	3	2	5	1	5	5	5
3	4	4	1	2	3	1	3	5	5
4	3	4	2	0	0	2	2	5	4
5	3	3	3	4	4	3	2	5	5
6	4	4	2	1	4	2	0	4	4
7	3	5	3	4	4	4	4	4	4
8	5	5	2	1	5	1	1	5	5
9	4	5	2	2	4	1	0	4	4
10	4	4	2	3	4	2	5	4	5
11	4	4	2	4	5	2	4	4	4
12	4	5	2	4	4	2	3	4	4
13	5	4	1	1	5	2	0	4	5
14	3	4	3	0	4	2	2	5	5
15	2	4	2	3	0	2	3	3	2
16	2	3	2	5	5	1	3	5	5
17	4	5	2	2	5	1	0	5	5
18	3	3	4	4	3	1	3	5	5
19	4	3	2	1	3	1	4	5	3
20	5	2	2	2	5	2	4	5	5
21	4	5	2	2	4	1	4	4	4
22	3	3	2	2	4	3	1	3	3
23	4	5	1	2	5	5	0	5	5
24	3	5	2	2	3	2	2	4	4
25	4	4	2	2	4	2	3	3	3
26	3	5	2	2	5	1	4	5	3
27	2	2	3	2	3	3	3	5	5
28	3	5	2	2	3	2	4	5	3
29	4	4	3	2	5	2	4	5	5
30	4	5	1	3	4	3	0	4	5
31	5	4	2	4	5	2	4	0	5
32	5	5	1	1	5	1	5	5	5
33	5	5	2	2	5	2	4	5	5
34	3	4	3	1	3	1	4	4	3
35	3	4	2	2	4	2	3	4	4
36	3	2	3	2	4	2	1	4	2
37	4	5	1	2	4	1	4	4	4
38	5	4	4	4	3	2	3	4	5
39	5	5	2	2	4	1	5	5	4
40	5	5	1	1	4	1	5	5	4
41	3	2	2	2	0	1	0	3	4
42	4	2	2	1	0	2	3	3	4
43	4	4	0	0	3	2	0	0	4
44	2	3	3	4	5	1	4	0	5
45	2	5	1	2	3	3	2	3	3
46	2	3	2	2	4	2	4	4	4
47	4	5	2	1	4	1	4	5	4
48	5	5	2	1	4	1	4	5	5
49	4	5	2	2	4	2	0	5	4

50	2	3	2	2	4	1	5	5	4

PS_10	PS_11	SS_UNZ_1	SS_UNZ_2	SS_UNZ_3	SS_UNZ_4	SS_UNZ_5	SS_UNZ_6	SS_UNZ_7	SS_UNZ_8
1	5	1	1	1	1	1	1	1	1
2	3	2	1	1	1	1	1	1	2
2	5	1	1	1	1	4	4	1	1
2	4	1	1	1	1	1	1	1	1
5	2	1	2	1	1	1	1	1	2
2	4	2	2	1	2	2	2	2	2
3	4	1	2	2	2	2	3	2	2
1	5	1	2	1	1	2	2	1	2
2	4	1	2	1	2	2	2	2	2
5	4	2	3	2	2	2	2	3	1
2	4	2	2	1	1	2	2	2	2
2	4	2	2	1	1	2	1	1	2
2	5	1	1	1	2	1	1	2	2
2	5	0	0	1	2	2	0	2	0
3	3	2	2	2	1	2	2	3	0
2	4	2	2	1	2	2	4	2	4
3	5	1	2	1	1	2	2	2	2
2	4	2	3	2	2	2	2	2	2
4	3	2	4	1	1	2	2	1	2
1	4	2	2	1	2	1	2	1	2
1	4	1	1	1	1	1	1	1	1
3	2	1	4	1	2	3	2	2	3
2	5	1	2	1	1	1	1	1	2
3	4	2	3	1	2	1	0	3	3
2	4	2	2	2	2	2	2	2	2
2	4	1	2	1	1	2	2	2	2
3	5	2	3	2	3	2	2	2	2
3	3	1	2	1	2	1	1	2	2
2	5	1	1	1	1	1	1	1	1
2	4	2	2	1	1	2	2	2	2
2	5	1	2	1	2	2	2	2	2
2	5	1	1	1	1	1	1	1	1
2	5	1	1	1	1	1	1	2	2
2	4	1	2	1	1	1	2	2	1
2	4	2	2	1	1	1	2	2	2
5	4	1	2	1	2	2	2	2	4
2	4	1	2	1	2	2	2	2	2
3	4	2	4	2	3	2	2	3	3
2	5	2	2	2	2	2	2	2	2
1	5	1	1	1	1	1	1	1	1
3	3	1	2	1	1	1	2	2	2
2	4	1	1	1	1	1	1	1	1
2	4	1	1	0	1	2	1	1	1
1	5	2	3	0	2	4	4	3	3
3	3	1	1	1	1	2	2	1	1
4	4	2	3	2	2	1	2	2	2
2	5	1	2	1	2	1	1	2	2
1	5	1	1	1	1	2	2	0	1
1	4	1	2	1	1	2	2	2	2

5	4	1	4	1	1	4	4	1	3

SS_UNZ_9	SS_And_1	SS_And_2	SS_And_3	SS_And_4	SS_And_5	SS_And_6	SS_And_7	SS_And_8	SS_And_9
1	1	1	1	1	1	1	1	1	1
1	3	3	4	4	4	4	4	4	4
1	2	2	2	1	4	4	1	1	1
1	1	2	1	1	1	1	2	1	1
1	2	2	2	2	2	2	2	2	2
1	2	2	2	3	2	2	2	2	2
2	2	2	2	2	3	3	3	3	2
1	2	3	1	1	3	3	2	1	1
2	2	2	2	2	2	2	2	2	2
2	3	4	2	3	2	2	2	2	3
1	2	2	1	2	2	2	2	2	2
1	2	2	1	1	2	2	2	2	1
1	2	2	2	2	2	2	2	2	2
0	1	0	0	0	2	0	2	0	0
2	2	2	2	1	2	2	2	0	2
2	2	2	1	2	2	4	2	4	2
2	2	2	2	2	2	2	2	2	2
2	2	2	1	2	2	2	2	1	2
2	2	2	2	2	2	2	2	2	2
2	3	3	3	4	3	3	4	4	4
1	2	2	2	2	2	0	2	1	2
1	3	3	2	3	3	2	3	2	3
1	2	2	1	1	2	2	2	2	1
3	3	3	2	4	3	0	3	0	4
2	2	2	2	2	2	2	2	2	2
1	2	3	2	2	3	3	4	3	3
2	3	3	2	3	3	3	3	2	2
2	2	2	1	2	2	1	2	2	2
1	1	2	1	2	1	2	2	2	1
3	3	4	3	4	4	4	4	0	4
2	2	2	2	3	2	2	2	2	2
1	1	1	1	1	1	1	1	1	1
1	2	1	1	2	2	2	2	1	1
2	4	3	1	1	2	2	4	2	3
1	2	2	1	2	2	2	2	2	2
1	1	2	2	4	4	4	2	3	1
1	2	2	2	2	2	2	2	2	2
2	3	3	2	3	3	2	2	2	3
2	2	2	2	2	2	2	2	2	2
1	2	2	1	1	2	2	2	1	1
2	1	2	1	2	2	2	2	2	2
1	2	2	2	2	2	2	0	2	2
1	1	1	0	1	2	1	1	1	1
2	4	3	1	1	2	3	3	2	2
1	1	2	1	1	2	2	1	1	1
4	2	2	2	2	2	2	2	2	2
2	2	2	2	3	2	2	2	2	2
1	2	2	1	1	3	3	3	3	1
1	3	3	2	3	2	2	3	2	2

1	4	4	1	1	4	4	4	3	1

Unh_UNZ_	Unh_UNZ_	Unh_UNZ_	Unh_UNZ_	Unh_UNZ_	Unh_UNZ_	Unh_and_1	Unh_and_2	Unh_and_3	Unh_and_4
1	1	1	1	1	1	1	1	1	1
2	2	1	2	2	2	4	4	4	4
1	1	1	1	1	1	1	1	1	1
1	1	1	1	1	2	2	1	1	1
2	2	2	2	1	1	2	2	2	2
2	2	2	2	2	2	2	2	2	2
2	2	2	2	1	2	3	4	2	2
1	1	1	1	1	1	1	2	1	2
1	2	1	1	2	2	2	2	1	2
2	3	1	1	2	2	3	4	1	1
2	2	2	2	1	2	3	2	2	2
2	2	1	2	2	2	2	2	1	2
1	2	1	1	1	1	3	2	1	2
1	3	0	1	4	2	1	2	2	1
2	0	2	2	0	0	3	0	0	0
2	2	2	1	1	2	3	3	3	2
2	2	2	2	2	2	2	2	2	2
2	2	2	2	1	2	2	1	1	2
2	2	1	1	1	2	2	3	1	3
2	1	1	1	1	1	2	3	2	2
2	2	1	1	3	3	3	4	1	1
1	1	1	1	1	1	1	1	1	1
2	3	0	2	3	3	3	4	2	2
2	2	2	2	2	2	2	2	2	2
3	3	2	3	4	3	4	3	2	3
1	1	1	1	2	2	1	2	1	1
1	1	1	1	1	1	1	1	1	1
2	2	2	2	3	3	3	3	3	3
1	2	1	2	1	1	2	2	0	2
1	1	1	1	1	1	1	1	1	1
2	2	1	1	1	2	3	3	1	2
2	2	1	1	1	2	2	2	1	1
2	2	2	2	2	2	2	3	1	2
2	2	1	1	2	2	2	2	2	1
2	2	2	1	2	2	3	4	3	1
2	2	2	2	2	2	3	3	3	3
2	2	1	1	1	1	2	2	2	2
2	2	1	1	1	1	2	3	1	1
2	1	1	1	1	2	2	2	2	2
1	1	1	1	1	1	1	1	1	1
2	2	1	3	3	2	3	2	1	3
1	2	1	1	2	1	2	2	1	2
2	2	2	2	3	3	3	3	3	2
2	2	1	1	2	1	3	2	2	1
1	1	1	1	1	1	1	2	1	1
1	1	0	1	1	2	2	2	0	1

1	1	1	1	1	1	2	1	2	2

Unh_and_!	Unh_and_(	Ger_1	Ger_2	Ger_3	Ger_4	Ger_5	Ger_6	Ger_7	Ger_8
1	1	6	6	7	7	7	7	6	6
4	5	5	3	3	2	7	3	5	7
1	1	7	7	7	7	7	6	6	6
2	1	6	6	7	7	7	7	5	7
1	1	1	3	6	1	6	6	6	3
2	2	6	5	6	6	7	7	6	6
3	4	6	4	6	7	6	4	5	6
1	1	7	7	7	7	7	7	7	7
2	2	4	5	6	6	7	7	6	7
3	3	4	3	5	3	6	6	6	5
2	3	6	6	6	6	7	7	6	5
2	2	7	7	7	7	7	6	7	7
1	2	3	4	6	6	5	6	5	3
2	2	6	6	5	6	7	6	4	5
0	0	6	6	7	6	7	4	6	6
2	2	6	6	6	7	7	7	6	7
1	2	5	4	5	4	5	4	4	7
1	1	6	6	7	7	7	7	7	6
4	3	6	6	5	5	7	6	5	6
2	2	7	6	5	6	6	6	5	7
3	3	1	1	5	3	6	6	6	2
1	1	3	3	6	3	6	5	6	6
4	4	5	2	6	4	3	2	3	6
2	2	5	5	4	5	5	5	4	5
3	3	4	4	4	4	4	4	4	5
2	2	5	5	6	7	6	6	6	6
1	1	7	7	7	7	7	7	7	7
3	3	5	5	5	6	7	6	6	6
2	2	5	5	3	6	6	5	4	5
1	1	3	3	5	6	7	6	6	6
1	3	4	4	7	5	7	4	5	7
2	2	6	5	6	7	7	7	6	7
3	3	6	6	7	7	6	7	6	6
2	2	6	6	6	7	6	6	6	6
2	4	3	3	3	2	6	5	4	5
3	3	6	6	7	6	7	7	7	7
2	2	7	5	4	7	3	3	3	3
1	2	6	6	7	7	7	7	7	7
1	3	5	5	6	5	6	7	5	4
1	1	7	6	7	7	6	7	7	6
2	2	5	6	7	7	7	6	6	7
2	1	4	2	4	5	5	4	4	4
3	3	6	6	6	7	4	4	4	5
2	2	3	6	6	6	6	7	6	7
1	1	3	5	4	6	7	6	6	5
2	2	5	4	6	4	7	6	6	6

2	1	6	6	7	7	6	7	7	5

Freq	Qual	Zweck_1	Zweck_2	Zweck_3	Zweck_4	Zweck_5	Zweck_NB	Situ_1	Situ_2
1	5	1							
2	5	1	2	3	4				
1	5	1		3	4				
1	4		2	3	4			1	
2	5			3					
1	5	1			4			1	2
4	3	1	2	3					
1	5	1							
2	4	1							
3	4	1		3	4			1	
2	3		2	3				1	
3	4	1							
1	5	1		3				1	
3	3	1		3	4			1	2
2	4	1		3				1	
2	5	1			4	5		1	
4	3				4				
2	5	1			4	5			
3	5	1	2	3	4			1	
3	4			3	4				
4	3	1		3	4				
2	5				4			1	
3	4			3					
1	4	1	2					1	2
3	4		2	3	4				
3	3	1		3	4	5		1	
1	5	1			4				
2	5				4				
3	4	1			4	5		1	2
6	4	1			4				
3	5		2			5			
3	4	1	2	3	4			1	
3	4				4	5			2
2	4	1						1	
2	4	1	2		4	5		1	2
3	5				4			1	
1	5	1							
3	5	1			4			1	
2	5	1		3	4				
6	4						6		
3	5	1			4	5			
1	5	1		3	4				2
3	4	1	2		4			1	2
3	3	1							
2	4	1			4				
4	5	1	2	3	4			1	

3 5

Situ_3	Situ_4	Situ_5	Situ_KA	Quelle	Quelle_UN	Quelle_anc	Klinik_1	Klinik_2	Klinik_3
			6	0	0	0	1	1	1
3		5		2	2	4	3	4	5
3		5		2	0	4	1	1	1
3		5		3	0	0	1	1	1
		5		2	2	4	12	11	6
		5		2	0	0	1	1	1
		5		2	0	4	7	3	5
3		5		2	0	4	3	9	5
		5		2	0	4	11	7	2
		5		2	2	2	11	1	1
		5		3	3	4	3	10	5
3		5		2	4	2	5	7	4
		5		2	0	0	1	1	1
		5		1	6	0	5	7	12
				3	0	0	8	12	10
		5		2	0	4	5	11	12
				0	3	0	1	1	1
		5		3	0	0	3	4	5
3		5		2	6	2	5	4	2
3		5		2	0	2	5	10	1
		5		2	0	2	3	5	6
				2	2	2	4	5	1
3		5		3	6	4	7	2	5
				3	0	0	1	1	1
3		5		2	2	4	1	1	1
		5		3	0	0	10	9	8
			6	2	0	4	1	1	3
		5		2	6	2	4	7	2
		5		3	0	2	1	1	1
		5		0	0	0	1	1	1
3		5		2	0	4	3	10	1
3		5		3	2	2	5	4	3
		5		3	2	4	3	7	12
		5		2	0	0	5	4	1
	4	5		2	2	4	3	9	10
		5		2	6	2	11	1	1
		5		2	6	4	11	12	5
		5		2	0	0	1	1	1
		5		2	0	4	3	2	6
			6	3	6	6	1	1	1
		5		2	2	4	6	10	1
		5		2	2	4	7	11	5
3		5		2	2	4	5	11	4
3		5		3	0	4	5	9	11
3				2	0	1	5	7	1
		5		2	6	4	1	1	1

5	2	2	4	11	1	1

Fkt	Dienstalter	Alter	Geschlecht
