# Supplementary material for: Bad manners in the Emergency Department: Incivility among doctors
Source: PLoS One. 2018 Mar 29;13(3):e0194933. doi: 10.1371/journal.pone.0194933 (PMC5875803; doi:10.1371/journal.pone.0194933)
Supplement: S3 File — (PDF) [file pone.0194933.s003.pdf]

Incivility among doctors

Social interaction in the emergency department

How do you assess the following aspects?

	Not correct	Rather not correct	undecided	Rather correct	correct	Not evaluable
1. Team members can openly admit their problems						
1. No one in this team would deliberately act in a way that undermines my efforts.						
2. If you make a mistake, it is often held against you						
3. People in this team sometimes dismiss others for being different						
4. In this team, my personal skills and talents are valued and utilized.						
5. It is difficult to ask other team members for help						
6. In this team it is safe to take a risk						
7. My superiors often encourage me to show initiative						
8. My superiors are interested in knowing my ideas and suggestions						
9. In the eyes of my superiors my opinion is less valued than theirs						
10. My superiors express that they appreciate my ideas and suggestions.						

Incivility among doctors

How correct are the following statements about the cooperation between medical colleagues?

In the emergency department?

	Not correct	Rather not correct	undecided	Rather correct	Correct	Not evaluable
1. There is often conflict between colleagues						
2. Some colleagues are unpleasant staff members						
3. You are irritated by every little thing						
4. There is just critique, no positive encouragement						
5. You have to work with mean colleagues						
6. You have to work with colleagues who cannot take a joke						
7. There are difficulties with cooperation between colleagues						
8. You have to take responsibility for others' mistakes						
9. If there is a mistake, some colleagues never start with themselves						

With other clinics/departments

	Not correct	Rather not correct	undecided	Rather correct	Correct	Not evaluable
1. There is often conflict between colleagues						
2. Some colleagues are unpleasant staff members						
3. You are irritated by every little thing						
4. There is just critique, no positive encouragement						
5. You have to work with mean colleagues						
6. You have to work with colleagues who cannot take a joke						
7. There are difficulties with cooperation between colleagues						
8. You have to take responsibility for others' mistakes						
9. If there is a mistake, some colleagues never start with themselves						

Incivility among doctors

How often a medical colleague…

…in the emergency department…

	Never	Rarely	Sometimes	Often	Mostly	Not evaluable
a) Put you down or was condescending to you?						
b) Paid little attention to you or showed little interest in your opinion?						
c) Made demeaning or derogatory remarks about you?						
d) Addressed you in unprofessional terms, either publicly or privately?						
e) Ignored or excluded you from professional camaraderie?						
f) Doubted your judgement on a matter which you have responsibility?						

…from another clinic/department…

Never	Rarely	Sometimes	Often	Mostly	Not evaluable

a) Put you down or was condescending to you?

b) Paid little attention to you or showed little interest in your opinion?

c) Made demeaning or derogatory remarks about you?

d) Addressed you in unprofessional terms, either publicly or privately?

e) Ignored or excluded you from professional camaraderie?

f) Doubted your judgement on a matter which you have responsibility?

Incivility among doctors

Own well-being

Do you agree with the following statements?

	Completely true	Mostly true	Partly true	Moderate true	Partly not true	Mostly not true	Not true	Not evaluable
I struggle to relax after work								
I think about work problems at home								
If others approach me, it I react grumpily								
I think about work problems on days off								
Sometimes I feel like a nervous wreck								
I am easily upset								
I react irritably even if I don’t wish to								
When I come home from work tired, I cannot relax								

Incivility among doctors

Frequency, nature, purpose and situation of incivility

How often appears incivility between medical colleagues in the emergency department?

rarely/never

few (once quarterly)

occasional (once a month)

Frequent (once a week)

Very often (daily)

Not evaluable

Compared to other clinics/departments how do you assess the quality of communication between doctors within the Emergency Department?

Much worse

Worse

Equal

Better

Much better

Not evaluable

What do you think is the purpose of incivility? (multiple answers possible)

Kommentiert [KWK1]: Cave wir haben einen punkt mehr in den abblidungen warum?

Unload stress

Leaving the emergency department as scapegoat

Shifting responsibilities

Power demonstration

Display of antipathy

Not evaluable

Other, following:

In which situation does incivility mostly appear? (multiple answers possible)

Acute situations (e.g. Resuscitation)

Handover inside ED

Admission to ward

Referral to external provider

Specialist consultation

No statement

Another possible situation, following:

Where does incivility mainly originate from?

Mainly from emergency department

Mainly from other clinics

Distributed equally

Other, following:

Incivility among doctors

Main source for incivility

Which grade is mostly responsible for incivility?

	Chief/Head physician	Senior physician	Deputy senior physician	Assistant physician	Student	Nurse	No statement
**In the emergency department**							
**In other clinics/departments**							

Comment:

Which other clinics are mostly responsible for incivility? Please fill in the 3 most common.

**1.**	**Most common:**	
**2.**	**Second most:**	
**3.**	**Third most common:**	

Comment:

Incivility among doctors

Personal information and general conclusions

The following information aim for a differentiated assessment. There won’t be a conclusion about individuals.

Your function

- Chief/Head physician
- Senior physician
- Deputy senior or assistant physician
- Student
- No statement

Number of years of service

Age

- <30 years
- 30 to 39 years
- 40 to 49 years
- >49 years
- No statement

Sex

- female
- male
- No statement

General statement about the questionnaire
